# Effect of a Novel Sugar Blend on Weight and Cardiometabolic Health among Healthy Indian Adults: A Randomized, Open-Label Study

**DOI:** 10.3390/foods11223545

**Published:** 2022-11-08

**Authors:** Srinath Aswathiah, Sunil Kumar Prabhu, Ramanna Lingaiah, Anusha Ramanna, Jyothi S. Prabhu, Shashi Kishor Pankaj, Arti Mehta, Arohi Bapna, Govindarajan Raghavan

**Affiliations:** 1 BGS Gleneagles Global Hospitals, Bangalore 560060, India; 2 Sarani Bio-Integra Private Limited, Bangalore 560062, India; 3 Tirumala Healthcare Limited, Bangalore 560085, India; 4 St. Johns Research Institute, Bangalore 560034, India; 5 Zydus Wellness R&D Centre, Zydus Wellness Institute, S.G. Highway, Off Ambli-Bopal Road, Ahmedabad 380058, India

**Keywords:** noncommunicable diseases (NCDs), body mass index (BMI), obesity, steviol glycosides (SGs), stevia, weight loss

## Abstract

Obesity is one of the major factors contributing to noncommunicable diseases (NCDs), which is associated with a high intake of a sugar-rich diet. Sugar blend (a novel combination of sugar and stevia) has half the calories of sugar with the same sweetness at recommended use and offers better compliance. A randomized controlled trial was conducted to evaluate the efficacy and safety of this sugar blend in normal to mildly overweight subjects with a body mass index (BMI) of 23–26 kg/m. Sixty subjects were categorized into Group A: Sugar group (n = 30), and Group B: Sugar blend group (n = 30). The primary outcomes evaluated were weight, waist circumference, hip circumference, waist/hip ratio, BMI, and the secondary outcomes evaluated were lipid profile, random blood sugar, and HbA1c. All these parameters were assessed at baseline, 30 days, 60 days, and 90 days. Group B showed a significantly higher weight loss (*p* = 0.013) at 90 days compared with Group A. A significant reduction in waist circumference (*p* < 0.0001) by 4.4 cm was noted at 90 days, in addition to reduction in total cholesterol (*p* < 0.0001), triglyceride (*p* = 0.006), LDL cholesterol (*p* = 0.0490), and VLDL cholesterol (*p* = 0.006) in Group B compared with the baseline. The study revealed that the sugar blend is an effective formulation in reducing weight, anthropometric factors, and other related metabolic parameters. It has been proven to be well tolerated and promotes weight loss when used in conjunction with a daily balanced diet and exercise plan.

## 1. Introduction

Obesity is caused by an increase in the consumption of high-fat, high-sugar energy-dense meals and decreased physical activity caused by the increasingly sedentary nature of many types of jobs, changing modes of transportation, and rising urbanization, as well as a lack of supportive policies in sectors such as health, agriculture, and others [1]. The consumption of sugar and sweets has been prominent in Indian culture, rituals, and religion [2]. People in India use roughly 25.17 kg of sugar per person/year, which is more than the worldwide per capita consumption of 23.7 kg per year [3]. Sweets and beverage consumption patterns among children are also changing fast [2]. A study revealed cola (sugar-sweetened beverages (SSBs)) consumption patterns among children and adolescents in urban India, with children and adolescents consuming about 1.8 cans of cola per week (540 mL/week; 1 can or 300 mL = 132 kcal and 33–40 g sugar), which might result in almost 1.3 kg of weight gain per child per year [2,4]. India’s rising sugar consumption per capita is a major problem and thus requires immediate attention [2]. The 66th round of the National Sample Survey Office (NSSO) 2009–2010 study indicated that roughly 13.2 teaspoons of white sugar were used per person/day in India in 2010 (55.3 g/day), up from 22 g/day in 2000 [5]. These values exceed the ones suggested by the National Institute of Nutrition’s Dietary Guidelines for Indians (2011), according to which healthy individuals should consume no more than 20–25 g of added sugar per day [6]. India is facing a potential healthcare crisis, owing to rising obesity rates and a significant shift in disease burden to NCDs, including cardiovascular disease (CVD), diabetes, hypertension, cancer, kidney disease, chronic respiratory disease, and nonalcoholic fatty liver disease [7,8,9]. According to the National Nutrition Monitoring Bureau (NNMB) 2016–2017 survey conducted among the urban population in India, 34% of men and 44% of women were overweight and obese. Further, about 22.3% of men and 22.4% of women had high cholesterol levels, while the overall prevalence of hypertension was found to be 31.1% and 26.1% among men and women, respectively [10]. A population-based cross-sectional study conducted in 15 states of India by the Indian Council of Medical Research–INdia DIABetes (ICMR-INDIAB) showed the overall prevalence of diabetes to be 7.3%, while that of prediabetes was 10.3% [11].

According to the World Health Organization (WHO) 2015 guidelines, free sugar intake should be decreased to less than 10% of total calorie intake in both adults and children [12]. In light of this remark, a range of measures targeted at lowering free sugar intake have been recommended, with food reformulation being one of the most notable options [13]. Regulating the calorie density of meals might be a revolutionary way to effective bodyweight loss by reducing energy consumption [14,15]. Sugar substitutes or low-calorie sweeteners (LCS) help lower the daily sugar and calorie intake. These dietary modifications can help in glycemic, weight, and cardiometabolic control [16]. Low-calorie sweeteners are employed in minute amounts to bestow the necessary degree of sweetness, while providing very little or no energy to the final product due to their strong sweetening power relative to sugars. These are simple approaches to cutting calories and sugars from our diet without compromising the enjoyment of sweet foods and beverages. Furthermore, because such calorie reductions are accomplished without a change in total dietary sweetness or palatability, such “sugar swaps” are anticipated to result in increased dietary compliance and better weight management results for individuals in the long run [14]. Natural LCS have gained immense interest in the toxicological acceptability and commercial development of steviol glycosides (SGs) [17]. Steviol glycoside, derived from *Stevia rebaudiana* Bertoni, provides no calories, and antioxidant properties, and is heat/pH stable [18,19,20]. When compared with sucrose, stevia is approximately 100 to 300 times sweeter [21]. Several Generally Recognized as Safe (GRAS) notices are received by the U.S. Food and Drug Administration (FDA) for the use of high-pure (at least 95% purity) SGs, among others, to which the FDA has no objection under the intended circumstances of use [22]. In addition, the acceptable daily intake (ADI) of 0–4 mg/kg BW, represented as steviol, was validated by the Joint Food and Agriculture Organization (FAO)/WHO Expert Committee on Food Additives [23].

People with conscious mindsets often try cutting down sugar or replacing sugar solely with LCS; however, compliance is the challenge. Sugar blend is a novel product manufactured with sugar and stevia combination that is 50% lower in calories when used as directed. It looks like crystal sugar and gives the same sweetness with half the amount of sugar. Stevia contributes to zero calories [17]. As a result, the use of sugar is reduced by 50%, and because it resembles sugar, it offers better compliance. This product lowers calorie consumption, which is linked to weight loss that in turn is associated with the prevention of diseases such as CVD. Moreover, it is an effective alternative for people who are habituated to high sugar intake with an insight into a healthy lifestyle, i.e., reduce calorie intake to maintain weight, yet desire the same appearance product as sugar. With this perspective, the current study was conducted to evaluate the efficacy and safety of completely replacing table sugar with sugar blend in the diet while maintaining a healthy lifestyle in healthy adults with a BMI of 23–26 kg/m^2^.

## 2. Material and Methods

### 2.1. Study Design

This was a randomized, open-label, two-arm, active-controlled, clinical trial.

### 2.2. Study Population

Over 60 subjects between the ages of 18 and 50 years were included in the study. The subjects were categorized into two groups:

- Group A: Sugar group (n = 30), who consumed ordinary table sugar (sucrose) (Active, control group), and
- Group B: Sugar blend group (n = 30), who substituted ordinary table sugar with half the quantity of sugar blend in their diet (Test group).

Inclusion criteria:

1. Normal to preobese participants (BMI = 23 kg/m^2^ to 26 kg/m^2^).
2. Subjects who are willing to provide written informed consent.
3. Those who agree to follow the general dietary, sugar intake, and exercise guidelines.
4. Subjects who have not taken part in a comparable study in the previous four weeks.

Exclusion criteria:

1. Subjects who have hypertension, renal, hepatic, or cardiac failure.
2. Endocrine diseases, such as hypothyroidism (if already on treatment).
3. Subjects with dyslipidemia or fat metabolic inborn errors.
4. Subjects who have postprandial glucose of more than 150 mg/dL in two tests and are considered early diabetics.
5. Any history of medication hypersensitivity or an adverse reaction that might affect the study.
6. Antidiabetic medicines such as metformin and statins, as well as nutritional supplements that may affect body weight, body fat, or blood cholesterol levels.
7. Women who are pregnant or lactating.
8. Refusal to sign an informed consent form.

### 2.3. Study Protocol

In the sugar group, the subjects used regular table sugar in their diet. In the sugar blend group, the subjects were asked to replace regular table sugar with a standard amount of test product, i.e., sugar blend in beverages such as tea, coffee, smoothies, milk, and other food products, and the amount consumed was noted in the subject’s diary. The spoons given to the sugar group dispensed approximately 5 g, whereas the spoons given to the sugar blend group dispensed around 2.5 g product.

Additionally, subjects were asked to restrict their daily diet to 2300 kcal/day (for women) and 2500 kcal/day (for men) and record their daily food intake in diet diaries. During follow-ups, the diet diary was checked to ensure compliance, and subjects were asked to adhere to the above kcal restriction. The subjects were asked to follow a moderate-intensity exercise routine, either 45 min of brisk walking or gym exercises daily for at least 6 days a week.

The study documentation, including the study protocol, was approved by the Institutional Ethics Committee (IEC)-Lifeline Ethics Committee in a review meeting conducted in November 2019 and again in November 2020, and is registered with the Clinical Trial Registry of India (CTRI) under the reference number CTRI/2021/02/030921.

The analysis was done within the group for all parameters at baseline, 30 days, 60 days, and 90 days. In addition, the parameters in Groups A and B were compared at baseline, 30 days, 60 days, and 90 days.

#### 2.3.1. Exception

Any additional sugar in both groups was allowed only at the discretion of the subject, who wished to attend a function or party not exceedingly once every month. The additional sugar consumed was picked up from a tabulated diary. Small discretion during daily life was allowed if the planned sugar intake was noted and recorded. No additional sugar substitute of any other kind was allowed other than the test product.

#### 2.3.2. Primary Outcomes

The primary outcomes analyzed were changes in weight, waist circumference, hip circumference, and waist/hip (W/H) ratio, BMI, noted at baseline, 30 days, 60 days, and 90 days, respectively.

#### 2.3.3. Secondary Outcomes

Biochemical investigation of blood lipid profile (total cholesterol, triglyceride, low-density lipoprotein (LDL) cholesterol, and very-low-density lipoprotein (VLDL) cholesterol, high-density lipoprotein (HDL)), random blood sugar (RBS), hemoglobin A1c (HbA1c), and liver function test (serum glutamate pyruvate transaminase, serum glutamic oxaloacetic transaminase, gamma-glutamyl transferase, urea, serum creatinine) were conducted at start and end of the study. Blood lipids determination using the enzymatic colorimetry method and random sugar were measured at each visit on baseline, 30 days, 60 days, and 90 days.

#### 2.3.4. Safety Evaluation

The safety of the test product in Group B subjects was evaluated. The clinicians (investigators, i.e., medical doctors) assessed the safety by observing if there were any adverse reactions reported by any subjects.

### 2.4. Statistical Analysis

Continuous variables such as age and instrumental readings were reported using descriptive statistics (mean, standard deviation (SD), number of subjects (n), median, and range), whereas categorical variables such as gender, race, and other factors were characterized using count (n) and percentage (%) of subjects. All comparisons within and between groups were performed using two-tailed paired t-tests and independent sample t-tests with a significance threshold of 5%, respectively. Correlation coefficients between the primary and secondary parameters were also computed. All statistical analyses were performed using XLSTAT—2019 by Addinsoft LLC (Paris, France).

## 3. Results

The baseline characteristics of both groups are listed in Table 1.

### 3.1. Weight

Both Groups A and B had significant differences in body weight at 90 days compared with the baseline (*p* < 0.0001) (Table 2). In Group B, there was a significant weight reduction (*p* = 0.0004) at 60 days compared with the baseline (Table 3).

**Figure 1:**
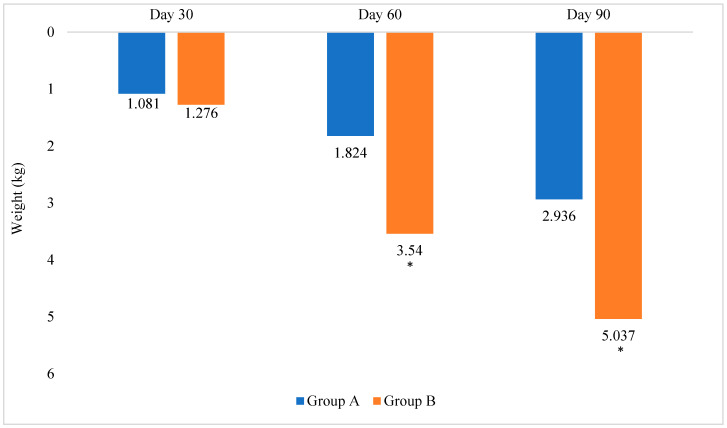
Change in weight (%) from the baseline. * *p* value significant depicting the comparison between Groups A and B.

Group B showed a higher weight loss compared with Group A (*p* = 0.013). At 90 days, Group B lost 3.393 kg (about 5% body weight) from the baseline, while Group A lost 1.897 kg (2.9% body weight) from the baseline (Figure 1) and (Table 3).

Group B lost approximately 1.7 times more weight than Group A at the end of 90 days, compared with the baseline.

### 3.2. Waist Circumference

Within Groups A and B, a significant difference was observed in waist circumference at 90 days compared with the baseline (*p* = 0.001; *p* < 0.0001, respectively). Group B had a mean waist circumference reduction of 4.4 cm after 90 days (Table 2). In Group B, there was also a significant reduction in waist circumference (*p* < 0.0001) at 60 days compared with the baseline (Table 3).

However, between Groups A and B, there was no significant difference in waist circumference at 90 days (Table 3).

### 3.3. Hip Circumference

Within both groups, a significant difference was observed in hip circumference at 90 days compared with the baseline (*p* < 0.0001). In Group B, a mean hip circumference reduction of 2.7 cm was observed at 90 days (Table 2), and a significant reduction in hip circumference (*p* < 0.0001) was observed at 60 days compared with the baseline (Table 3).

However, between Groups A and B, there was no significant difference in hip circumference at 90 days (Table 3).

### 3.4. Waist/Hip Ratio

Group B had a significant change in W/H ratio from baseline 0.882 ± 0.052 to 0.862 ± 0.045 at 90 days (*p* < 0.0001), whereas it was nonsignificant in Group A (Table 2).

There was no significant difference in the W/H ratio from baseline between Groups A and B at 90 days (Table 3).

### 3.5. Body Mass Index (BMI)

Between Groups A and B, there was no significant difference in the BMI values at 90 days. Within Groups A and B, a significant reduction in the BMI was observed at 90 days when compared to baseline (Table 2).

### 3.6. Secondary Outcomes

In within-group analysis, Group B showed a significant reduction in total cholesterol (*p* < 0.0001), triglyceride (*p* = 0.006), LDL cholesterol (*p* = 0.0490), VLDL cholesterol (*p* = 0.006), RBS (*p* = 0.028), and HbA1c (*p* < 0.0001) at the end of 90 days compared with the baseline. Between Groups A and B, there was no significant difference in, lipid profile, RBS, or HbA1c values. These blood parameters were within the clinical reference range (Table 4).

### 3.7. Safety Evaluation

Overall, the sugar blend was well tolerated and there were no dropouts or complaints in the study. No adverse events were reported during the trial period, and no adverse changes in biochemical secondary parameters (blood lipid profile, blood glucose levels) were seen. Hence, the product could be considered safe.

## 4. Discussion

People in India are at an elevated risk of metabolic disorders owing to a higher metabolic load. It is believed that for every given level of BMI, there is a proportionally higher ratio of fat to lean mass, and as BMI rises, the metabolic load rises too. Therefore, preventing excess weight gain, abdominal obesity, and sedentary behavior, as well as a transition to low-glycemic index and lower fat diets, are necessary [24]. A diet that can self-regulate dietary energy intake can play a key role in long-term weight loss, weight maintenance, and body weight gain prevention [15].

According to World Health Organization (WHO) 2015 guidelines, free sugar intake should be kept to less than 10% of total calorie intake in both adults and children [12]. Sugar restriction while retaining natural taste and appearance in products is a revolutionary concept that sugar blend has achieved with a balanced formulation of natural sweetener stevia and sugar in a proportion to produce the same sweetness of sugar at 50% consumption as instructed. Half the quantity of sugar blend, i.e., 2.5 g of sugar blend is equivalent to 5 g of table sugar in sweetness, thereby giving 50% lesser calories. The present study was conducted to enumerate the benefits of sugar blend in healthy adults.

WHO classified stevia as a natural sweetener in its noncaloric sweetener classification (i.e., natural and artificial) [25,26]. In the United States, it is widely recognized as a natural sweetener, and imagery and “natural” phraseology are utilized in many parts of the world to communicate to customers the usage of natural-origin plant-based stevia sweeteners [25]. The Food Safety and Standard Authority of India has approved steviol glycoside intense sweetener SG (INS 960) as a sweetener. The standard for this sweetener is defined in “The Food Safety and Standards (Food Products Standards and Food Additives) Regulations, 2011, Clause 3.2.2 (1)” [27].

In India, any celebration is traditionally marked by the consumption of sweets. After every meal, any happy event, religious holiday, social gathering, etc., it is a tradition to “sweeten the tongue” [2]. Excess sugar consumption leads to the buildup of body fat and intra-abdominal fat, increased insulin resistance, excess liver fat, hypertriglyceridemia, increased free fatty acids, hyperuricemia, and diabetes among other metabolic disorders [3]. According to a recent report by the Scientific Advisory Committee on Nutrition (SACN), there is some evidence that SSBs are associated with weight gain, as well as consistent evidence that they are linked to an elevated risk of type 2 diabetes mellitus [28]. Further, obesity, like other cardiovascular risk factors, contributes to the development of CVD and associated mortality [29]. Non-nutritive sweeteners can help people cut back on added sugars, resulting in calorie reduction and weight loss/control, as well as positive impacts on metabolic parameters. Nonetheless, if there is a compensatory increase in calorie intake from other sources, these potential advantages will not be completely realized [30]. The American Heart Association (AHA) and the American Diabetes Association (ADA) issued a joint statement saying that stevia and comparable sweeteners can help patients with diabetes provided they use them correctly and do not compensate by consuming more calories later in the day [30].

Overweight and obesity are caused by an energy imbalance between calories ingested and calories burned [1]. One pound of body weight is 3500 calories. This indicates that if a person cuts (or adds) 500 calories to the daily diet, they will lose (or gain) one pound every week, i.e., 500 calories each day multiplied by seven days equals 3500 calories [31]. Therefore, at least 45 min of moderate-intensity physical activity 5 days a week is also advisable. This level of physical exercise may lower the chance of developing some chronic conditions [6].

The current study aims to understand the effect of sugar replacement with blended sugar, which gives the same sweetness in half the amount. A mere cut down in sugar kcal, without compromising the sweetness, can help in improving metabolic parameters and weight loss. Here, when sugar was substituted with sugar blend, which resulted in consumption of just 50% sugars, the weight loss was 3.393 kg (*p* < 0.013) compared to 1.897 kg in the sugar group, indicating that there is a calorie reduction and weight loss, as well as a healthy strategy of losing weight with better compliance. The effect of confounding factors such as physical activity was also taken into consideration. There was a weight loss in the sugar group, which could be due to dietary restrictions and a moderate physical activity routine; however, more weight loss was observed in the sugar blend group, which could be due to physical activity along with the kcal deficit.

In addition, compared with the baseline, a significant mean reduction was noted in the sugar blend group in metabolic parameters at the end of the study (90 days) owing to weight loss that is related to a reduction in calories. The HbA1c levels dropped slightly, showing that the product helped in lowering blood sugar in conjunction with calorie reduction and subsequent weight loss. An increase in these cardiometabolic parameters denotes the risk factors for diseases such as CVD, diabetes, etc., and altering these parameters would help in disease prevention.

At 90 days, the sugar blend group had a significant waist circumference reduction of 4.4 cm (*p* < 0.0001) and hip circumference reduction of 2.7 cm (*p* < 0.0001) from baseline.

This is a free-living trial; simply substituting sugar with sugar blend, a similar-looking product with half the calories in everyday life, would help people stimulate their routine, which in turn is associated with better outcomes. Better compliance offered by the product will assist people in losing weight. Sugar blend was found to reduce 1.7 times more weight than the regular table sugar (no substitution) along with physical activity, which could be attributed to the consumption of half the amount of sugar blend, thereby reducing the overall caloric intake. Reduction in calorie intake along with increasing calorie expenditure and regular exercise could lead to an energy deficit. It thus helped in weight loss, which in turn plays a remarkable role in CVD prevention.

The test product was found to be safe. In addition, the safety of stevia is established in the literature [23,32]. The Joint FAO/WHO Expert Committee has declared stevia to be safe [23].

Being an open-label, free-living community trial, there is a possibility that the results could have been influenced by the nature of the treatment and participant behavior. Although the sugar blend has a positive influence on body weight and other cardiometabolic parameters, the small sample size and possible ambiguity in the results due to participants’ personal choices necessitate further research on other metabolic parameters under a controlled environment.

## 5. Conclusions

Indians are at an elevated risk of metabolic disorders owing to higher metabolic load. It is believed that there is a relatively higher ratio of fat to lean mass for any given level of BMI. With an increase in BMI, the metabolic load also increases. In this clinical trial, we observed that among healthy adults, replacing regular/ordinary table sugar with novel products such as sugar blend (a combination of sugar and stevia) provides the same sweetness as sugar and better compliance. Half the quantity, e.g., 2.5 g of sugar blend is equal in sweetness to 5 g of sugar, resulting in 50% fewer calories compared with table sugar. When used in conjunction with a daily balanced diet and exercise routine, the sugar blend can help reduce weight, waist circumference, hip circumference, and other metabolic parameters. Overall, sugar blend was proven to reduce 3.39 kg weight, 4.4 cm waist circumference, and 2.7 cm hip circumference after 90 days when used as part of a daily balanced diet and regular exercise. Sugar blend, when used in conjunction with a daily balanced diet and exercise routine, helped people lose 1.7 times more weight than ordinary sugar. In addition, the product was found to be well tolerated. Such sugar blends provide a meaningful way to dietary control of metabolism and weight control, and larger population studies in the future will gather momentum to provide this for use by the population at large.

## Figures and Tables

**Table 1:** Baseline characteristics of both groups.

	Group A	Group B	*p* Value *
Total subjects (n)	30	30	
Male	18	17	
Female	12	13	
Aged 30 years or below	13	15	
Aged between 31 and 39 years	11	6	
Aged 40 years and above	6	9	
Weight (kg) ^†^	64.963 ± 9.861	66.993 ± 8.308	0.392
Waist (cm) ^†^	88.533 ± 7.637	88.567 ± 6.755	0.986
Hip (cm) ^†^	97.333 ± 7.373	99.867 ± 5.07	0.126
Waist/hip ratio ^†^	0.905 ± 0.058	0.882 ± 0.052	0.118
BMI (kg/m^2^) ^†^	24.01 ± 2.271	24.673 ± 2.023	0.204
Total cholesterol (mg/dL) ^†^	176.3 ± 23.476	187.80 ± 32.964	0.125
Triglyceride (mg/dL) ^†^	159.167 ± 70.445	177.733 ± 84.736	0.360
HDL cholesterol (mg/dL) ^†^	36.667 ± 5.862	38.567 ± 5.952	0.218
LDL cholesterol (mg/dL) ^†^	107.867 ± 17.795	113.64 ± 31.73	0.388
VLDL cholesterol (mg/dL) ^†^	31.833 ± 14.089	35.58 ± 16.929	0.355
RBS (mg/dL) ^†^	95.567 ± 17.561	93.10 ± 14.456	0.555
HbA1c % ^†^	5.53 ± 0.447	5.57 ± 0.462	0.735

BMI: Body mass index; HDL: High-density lipoprotein; LDL: Low-density lipoprotein; VLDL: Very-low-density lipoprotein; RBS: Random blood sugar; HbA1c: Hemoglobin A1c. ^†^ All values are presented as mean ± standard deviation. * *p* values significant depicting baseline comparison between Groups A and B.

**Table 2:** Change in weight, waist circumference (cm), hip circumference (cm), and waist/hip ratio within the group at 90 days.

	Group A			Group B		
	Baseline	Day 90	*p* Value	Baseline	Day 90	*p* Value
Weight in kg (mean ± SD)	64.963 ± 9.861	63.067 ± 9.791	<0.0001 *	66.993 ± 8.308	63.600 ± 8.071	<0.0001 *
Mean reduction in kg (%)		−1.897 (2.936)			−3.393 (5.037)	
Waist Circumference in cm (mean ± SD)	88.533 ± 7.637	84.633 ± 9.779	0.001 *	88.567 ± 6.755	84.167 ± 6.385	<0.0001 *
Mean reduction in cm (%)		−3.90 (4.462)			−4.40 (4.931)	
Hip circumference in cm (mean ± SD)	97.333 ± 7.373	94.900 ± 6.599	<0.0001 *	99.867 ± 5.070	97.133 ± 4.897	<0.0001 *
Mean reduction in cm (%)		−2.433 (2.441)		-	−2.733 (2.703)	
Waist/hip ratio (mean ± SD)	0.905 ± 0.058	0.900 ± 0.05	0.495	0.882 ± 0.052	0.862 ± 0.045	<0.0001 *
Mean reduction (%)		−0.005 (0.389)			−0.02 (2.24)	
BMI in kg/m^2^ (mean ± SD)	24.01 ± 2.271	23.323 ± 2.461	<0.0001 *	24.673 ± 2.023	23.447 ± 2.301	<0.0001 *

SD: Standard deviation. * *p* values significant depicting the comparison with the baseline.

**Table 3:** Change in weight (kg), waist circumference (cm), hip circumference (cm), waist/hip ratio, and body mass index between groups.

Parameters	Baseline		Day 30		Day 60		Day 90	
	Group A	Group B	Group A	Group B	Group A	Group B	Group A	Group B
Weight (kg) ^†^	64.963 ± 9.861	66.993 ± 8.308	64.250 ± 9.669	66.160 ± 8.460	63.807 ± 9.838	64.583 ± 8.267	63.067 ± 9.791	63.600 ± 8.071
*p* value (between groups) *p* value (within group)	0.392		0.419		0.742 0.0004 **		0.819	
Weight difference (kg) from baseline ^†^			0.713 ± 0.606	0.833 ± 1.475	1.157 ± 0.716	2.410 ± 3.313	1.897 ± 1.739	3.393 ± 2.688
*p* value (between groups)			0.682		0.047 *		0.013 *	
Weight difference % ^†^			1.081 ± 0.922	1.276 ± 2.393	1.824 ± 1.297	3.540 ± 4.427	2.936 ± 2.771	5.037 ± 3.650
*p* value (between groups)			0.679		0.046 *		0.015 *	
Waist circumference (cm) ^†^	88.533 ± 7.637	88.567 ± 6.755	87.000 ± 7.579	86.833 ± 6.721	86.200 ± 7.595	85.967 ± 6.392	84.633 ± 9.779	84.167 ± 6.385
*p* value (between groups) *p* value (within group)	0.986		0.929		0.898 <0.0001 **		0.828	
Waist circumference difference (cm) from baseline ^†^			1.533 ± 1.358	1.733 ± 1.946	2.333 ± 1.882	2.600 ± 2.824	3.900 ± 5.744	4.400 ± 2.283
*p* value (between groups)			0.646		0.668		0.659	
Waist circumference difference % ^†^			1.724 ± 1.519	1.941 ± 2.150	2.626 ± 2.060	2.870 ± 3.197	4.462 ± 7.042	4.931 ± 2.432
*p* value (between groups)			0.654		0.727		0.731	
Hip circumference (cm) ^†^	97.333 ± 7.373	99.867 ± 5.070	96.167 ± 6.859	98.567 ± 4.876	95.700 ± 6.737	97.167 ± 4.800	94.900 ± 6.599	97.133 ± 4.897
*p* value (between group) *p* value (within group)	0.126		0.124		0.336 <0.0001 **		0.142	
Hip circumference difference (cm) from baseline ^†^			1.167 ± 1.642	1.300 ± 2.020	1.633 ± 2.748	2.700 ± 2.351	2.433 ± 1.995	2.733 ± 2.703
*p* value (between groups)			0.780		0.112		0.627	
Hip circumference difference % ^†^			1.156 ± 1.617	1.277 ± 1.983	1.608 ± 2.729	2.672 ± 2.237	2.441 ± 1.887	2.703 ± 2.588
*p* value (between groups)			0.797		0.104		0.656	
Waist/hip ratio ^†^	0.905 ± 0.058 *	0.882 ± 0.052	0.899 ± 0.046	0.878 ± 0.050	0.896 ± 0.053	0.880 ± 0.048	0.900 ± 0.050	0.862 ± 0.045
*p* value (between groups)	0.118		0.098		0.244		0.003^*^	
Waist/hip ratio difference from baseline ^†^			0.005 ± 0.034	0.004 ± 0.016	0.009 ± 0.037	0.002 ± 0.027	0.005 ± 0.037	0.020 ± 0.023
*p* value (between groups)			0.809		0.387		0.052	
Waist/hip ratio difference % ^†^			0.456 ± 3.648	0.388 ± 1.785	0.885 ± 3.972	0.119 ± 3.105	0.389 ± 4.049	2.240 ± 2.524
*p* value (between groups)			0.927		0.409		0.038 *	
BMI (kg/m^2^) ^†^	24.01 ± 2.271	24.674 ± 2.023	23.751 ± 2.259	24.376 ± 2.283	23.578 ± 2.311	23.822 ± 2.476	23.323 ± 2.461	23.447 ± 2.301
*p* value (between groups)	0.24		0.29		0.69		0.84	

BMI: Body mass index. ^†^ All values are presented as mean ± standard deviation. * *p* values significant depicting the comparison between Groups A and B (between-groups independent sample *t*-test). ** *p* values significant depicting the comparison between Group B and the baseline (within Group B paired *t*-test).

**Table 4:** Secondary outcomes.

Parameters	Baseline		Day 30		Day 60		Day 90	
	Group A	Group B	Group A	Group B	Group A	Group B	Group A	Group B
Total cholesterol (mg/dL) ^†^	176.300 ± 23.476	187.800 ± 32.964	179.900 ± 25.841	182.367 ± 28.695	175.533 ± 24.274	171.333 ± 35.856	179.667 ± 22.225	175.333 ± 28.585
*p* value (between groups) *p* value (within group)	0.125		0.728		0.597		0.515 <0.0001 *	
Triglyceride (mg/dL) ^†^	159.167 ± 70.445	177.733 ± 84.736	157.100 ± 68.364	154.667 ± 52.341	150.000 ± 61.630	151.167 ± 48.471	149.533 ± 58.819	150.500 ± 43.793
*p* value (between groups) *p* value (within group)		0.360		0.878		0.935		0.943 0.006 *
HDL (mg/dL) ^†^	36.667 ± 5.862	38.567 ± 5.952	38.067 ± 5.271	39.267 ± 5.375	37.767 ± 4.439	39.300 ± 5.522	38.633 ± 4.745	38.300 ± 4.801
*p* value (between groups)	0.218		0.386		0.241		0.788	
LDL (mg/dL) ^†^	107.867 ± 17.795	113.640 ± 31.730	110.500 ± 18.877	112.167 ± 26.019	107.633 ± 18.669	104.613 ± 26.833	110.633 ± 17.616	106.933 ± 25.377
*p* value (between groups) *p* value (within group)	0.388		0.777		0.615		0.514 0.0490 *	
VLDL (mg/dL) ^†^	31.833 ± 14.089	35.580 ± 16.929	31.467 ± 13.673	30.933 ± 10.468	29.333 ± 12.337	31.967 ± 13.895	30.373 ± 11.746	30.167 ± 8.789
*p* value (between groups) *p* value (within group)	0.355		0.866		0.551		0.939 0.006 *	
RBS (mg/dL) ^†^	95.567 ± 17.561	93.100 ± 14.456	90.467 ± 12.252	93.200 ± 16.606	89.333 ± 9.925	84.962 ± 19.622	89.967 ± 8.002	86.767 ± 16.400
*p* value (between groups) *p* value (within group)	0.555		0.471		0.281		0.341 0.028 *	
HbA1c % ^†^	5.530 ± 0.447	5.570 ± 0.462	5.527 ± 0.376	5.483 ± 0.424	5.537 ± 0.334	5.377 ± 0.387	5.520 ± 0.350	5.437 ± 0.420
*p* value (between groups) *p* value (within group)	0.735		0.677		0.092		0.407 <0.0001 *	

HDL: High-density lipoprotein; LDL: Low-density lipoprotein; VLDL: Very-low-density lipoprotein; RBS: Random blood sugar; HbA1c: Hemoglobin A1c. ^†^ All values are presented as mean ± standard deviation. * *p* values significant depicting the comparison between Group B and the baseline (within Group B paired *t*-test).

## Data Availability

The data presented in this study are available on request from the corresponding author.
